# HIV-Positive and HIV-Negative Women with Medicaid Have Similar Rates of Stillbirth and Preterm Birth

**DOI:** 10.1089/whr.2021.0068

**Published:** 2022-01-04

**Authors:** Kathryn D. Thompson, David J. Meyers, Yoojin Lee, Susan Cu-Uvin, Ira B. Wilson

**Affiliations:** ^1^ Department of Health Services, Policy, and Practice, Brown University School of Public Health, Providence, Rhode Island, USA.; ^2^ Warren Alpert School of Medicine, Brown University, Providence, Rhode Island, USA.; ^3^ Providence/Boston Center for AIDS Research (CFAR), Providence, Rhode Island, USA.

**Keywords:** preterm, stillbirth, disparities, Medicaid, HIV, pregnancy

## Abstract

***Introduction:*** Women living with human immunodeficiency virus (WLHIV) may face additional challenges and differential birth outcomes when compared with women without human immunodeficiency virus (HIV). There is limited research to date studying birth outcomes among a nationally representative sample of WLHIV. This study compares stillbirth and prematurity rates between HIV-positive (HIV+) and HIV-negative (HIV−) mothers in the Medicaid program.

***Methods:*** We used 12 years (2001–2012) of Medicaid Analytic eXtract data. We included Medicaid claims from the 14 states with the highest prevalence of HIV: California, Florida, Georgia, Illinois, Louisiana, Massachusetts, Maryland, North Carolina, New Jersey, New York, Ohio, Pennsylvania, Texas, and Virginia. Primary outcomes were stillbirth and preterm birth. We used logistic regression models adjusting for age, race, Medicaid coverage, eligibility, substance use, rurality, comorbidities, and state fixed effects to compare differences in rates for women with and without HIV.

***Results:*** Our study included a total of 33,078 HIV+ and 7,663,758 HIV− pregnancies from Medicaid enrollees between 2001 and 2012. The proportions of stillbirths and preterm births were higher for HIV+ when compared with HIV− mothers (0.9% vs. 0.7% and 8.0% vs. 6.6%, *p* < 0.0001). After adjusting for covariates, being HIV+ was not significantly associated with both stillbirth (odds ratio [OR]: 1.05) or prematurity (OR: 1.01). Black race was a strong independent predictor of both stillbirth and prematurity (OR: 1.99 and 1.51, *p* < 0.01). Rurality and substance abuse were not associated with either outcome.

***Conclusions:*** After adjustment for relevant covariates, maternal HIV infection was not associated with increased rates of stillbirth or preterm birth in the Medicaid program in the United States. It is imperative that we understand and eliminate the clinical, social, and contextual factors that are responsible for the strong association between black race and poor perinatal outcomes that we observe.

## Introduction

Almost a quarter of persons with human immunodeficiency virus (HIV) infection in the United States are women, and ∼25.5% of those are of child-bearing age (between 25 and 44 years).^1,2^ Estimates are that ∼5,000 women with HIV (WLHIV) deliver an infant each year^3^ and the birth rate for this age group is around 7%; making it critical to understand how to optimize pregnancy outcomes for these women. Advances in HIV prevention, research, and universal treatment of antiretroviral therapy (ART) have reduced mother to child transmission (MTCT) by 95% and have been recommended for use by HIV-positive (HIV+) pregnant mothers since the 1990s.^4,5^

The first protease inhibitor (saquinavir) was approved in December 1995, ushering in the era of modern ART (formerly called highly active antiretroviral therapy or HAART). However, despite advancements and wide adoption of ART use, HIV infection remains a chronic disease, and a potential risk factor for numerous complications of pregnancy and adverse pregnancy outcomes in developed and developing nations.^6–8^ Two important pregnancy outcomes to consider are stillbirth and preterm birth.

Stillbirth refers to intrauterine death and subsequent delivery of a developing infant that occurs after 20 weeks' gestation. Worldwide, the major causes of stillbirth are childbirth complications, post-term pregnancy, and maternal infections, including malaria, syphilis, and HIV.^9,10^ Approximately 24,000 stillbirths occur in the United States annually which represents 1% of all pregnancies.^11^ In the United States, factors associated with perinatal mortality include black race, maternal age >35, low socioeconomic status, maternal pre-existing conditions, and previous stillbirth.^12^ Only 9%–15% of all stillbirths are caused by infections, which include syphilis, toxoplasmosis, parvovirus B-19, and chorioamnionitis.^13^

HIV is not listed among the top causes of stillbirth and instead is “purported to cause” stillbirth.^13^ A systematic review published in 1998 found a strong association between maternal HIV, stillbirth, and miscarriage, or spontaneous abortion.^7^ Later studies that examined pregnant women living with human immunodeficiency virus (WLHIV) during the era of modern ART—but outside of the United States—had similar findings.^14,15^ A retrospective cohort study that used the US Nationwide Inpatient Sample found that in adjusted analyses intrauterine death was not significantly associated with HIV infection.^6^ Because stillbirth is so uncommon, large data sets are needed to validly address whether HIV increases the risk for stillbirth in the United States in the era of modern ART.

In contrast, preterm birth—the delivery of an infant before 37 weeks' gestation—is more common, occurring in 10% of US deliveries.^16^ Preterm birth is among the five leading causes of perinatal mortality in the United States along with birth defects, low-birth weight, and maternal pregnancy complications.^16^

Common causes of preterm birth include intrauterine and extrauterine infections,^17^ including urinary tract infections, vaginal infections, viral infections, and sexually transmitted diseases.^18^ Studies with HIV-negative (HIV−) comparators from before the era of modern ART found that HIV infection was associated with increased rates of preterm birth.^7, 19^ A more recent meta-analysis comparing HIV+, but ART-naive, to HIV− mothers found a strong association between HIV and preterm delivery.^20^

Studies with HIV− comparators from the modern ART era have also tended to show that HIV+ women have higher rates of preterm birth. A meta-analysis that included 52 studies showed increased odds of preterm birth that was relatively unaffected by the period studied (pre- or post-1995), region of the world, or exposure to ART.^21^ Studies from Botswana,^14^ Spain,^22^ Switzerland,^23^ British Columbia,^24^ Nigeria,^15^ and French Guiana^25^ have all showed an increased risk of preterm delivery for mothers with HIV, although one article from South Africa did not.^26^

One US article studied deliveries at two tertiary care centers between 2000 and 2007 and found a twofold increase in preterm birth for WLHIV^27^; consistent with two studies that used the US National Inpatient Sample^6,28^ and two that focused on the added effects of advanced maternal age.^29,30^

Proper adjustment for confounding is a challenge in all these studies, because HIV+ mothers are different from HIV− mothers in ways that are difficult to capture in analytic data sets. This is particularly true in the United States, where WLHIV are more likely to be black, have a history of substance use (SU), and be uninsured or covered by Medicaid than HIV− mothers.^6^ US studies that use inpatient samples only^6,28^ cannot capture prenatal care may poorly capture relevant comorbid conditions (CCs), including SU and smoking, and may have incomplete data on race.^28^

Other studies that do extensive primary data collection tend to enroll small more local populations with limited generalizability.^27^ To address these issues, we conducted a study of rates of stillbirth and prematurity in a nationally representative sample of pregnant women with Medicaid insurance coverage from 2001 to 2012, during the era of modern ART.

Limiting the sample to Medicaid is appropriate because most pregnant WLHIV in the United States are covered by Medicaid, and because Medicaid beneficiaries all must meet criteria for having low income and assets. This reduces (but does not eliminate) confounding related to socioeconomic status. In addition, because we use both inpatient and outpatient data, we have the ability to control for race, prenatal care visits, SU, smoking, and CCs. Our study aims to answer the following question: Are rates of stillbirth and preterm birth higher in women with HIV infection compared with women who do not have HIV infection?

## Methods

### Data

This analysis used the Centers for Medicare and Medicaid Services Analytic eXtract (MAX) Database from January 1, 2001 to December 31, 2012. MAX files are Medicaid administrative claims files for persons enrolled in Medicaid and the Children's Health Insurance Program (CHIP). These data are collected in individual state's Medicaid Management Information System (MMIS), reported to CMS on a quarterly basis, and are then transformed into a consistent national format for research purposes. The MAX data we used includes Medicaid claims from the 14 states with the highest prevalence of HIV: California, Florida, Georgia, Illinois, Louisiana, Massachusetts, Maryland, North Carolina, New Jersey, New York, Ohio, Pennsylvania, Texas, and Virginia.

We used International Classification of Diseases, Ninth Revision, Clinical Modification (ICD-9-CM) codes from hospital-level inpatient and outpatient claims to identify the outcomes we analyzed. Additional variables included personal-level demographics, characteristics, diagnosis codes, procedural codes, Medicaid coverage periods, and prescription drug information for each individual for the corresponding year of delivery. We used the 2010 Census to identify geographic region and determine rural residence at the county level.

### Study design and sample

For this retrospective cohort study, we utilized Inpatient Hospital claims within the MAX files that indicated a singleton or multiple birth. We compared two groups of pregnancy claims, HIV+ and HIV−, which occurred within the 12-year study period to determine the association between the two outcomes of interest and maternal HIV infection. We classified women in our sample as HIV+ or HIV− using a previously validated algorithm that identified HIV diagnosis codes across inpatient, other therapy (OT; outpatient), long term, and pharmacy claims.^31^ For a pregnancy claim to be included in the HIV+ cohort, we required that the HIV infection diagnosis of the mother occurred before the recorded delivery date or within 1 year after giving birth.

The HIV− cohort was selected from the remaining pool of Medicaid deliveries who did not meet the HIV+ definition. A total of 72,454 deliveries were excluded due to ICD-9-CM coding errors and 6,434 deliveries were excluded due to no specification of birth outcome. In addition, 3,450 women were coded as males and 19,370 were removed due to inadequate information to classify them as HIV+ or negative. Our analysis included only women 18 years of age and older. Our final cohort included 5,750,885 unique women and 7,696,836 deliveries: 7,663,758 HIV− and 33,078 HIV+.

### Variables

The MAX database contains up to seven diagnosis codes for each hospitalization. Data for the outcome measures and clinical characteristics were derived directly from the inpatient claims using associated ICD-9-CM codes. Coding to identify specific variables of interest consisted of diagnostic codes and procedural codes from the ICD-9-CM and Physicians' Current Procedural Terminology (CPT) codes. The dependent variables in this analysis were stillbirth (V27.1, V27.4, and V27.7) and preterm birth (644.21).

Independent variables in this analysis included the following demographic and clinical characteristics: maternal age, maternal race and/or ethnicity, year of birth, state of birth, Medicaid basis of eligibility (BOE), location (rural residence), number of prenatal care visits within 9 months before delivery, type of Medicaid coverage, SU, and CCs. This information was collected from the Personal Summary and OT files within the MAX database.

Age was divided into three subcategories (18–24, 25–34, and 35+) and was treated as a categorical variable in analyses. Race was also divided into five categories: white, black, Hispanic, other, and unknown. The basis of Medicaid eligibility status was subdivided as being an adult, living in poverty, and other. Location and rural residence were dichotomized as rural versus nonrural using the 2010 Census ZCTA to Zip Code Crosswalk.

CCs were identified as previously described^31^ and the number of individual conditions was classified as 0, 1, 2, 3, 4, or 5+ total conditions. Number of prenatal visits were listed as 0 visits, 1–3 visits, 4–6 visits, and 7+ visits. Prenatal visits and number of months on Medicaid coverage were collinear in sensitivity analyses so only the number of prenatal visits were included in the final analyses. Medicaid coverage characterized as Fee for Service coverage, or other (managed care, no coverage, and Medicare dual eligible). Substance abuse was stratified into two categories: any SU and tobacco use. Patient admission date was used as a proxy for delivery date and this delivery date was used to determine coverage type at time of delivery.

### Analyses

We first determined the frequencies of sociodemographic and clinical characteristics and compared HIV+ and negative mothers using chi square tests. Using total overall births as the denominator, stillbirth and preterm birth rates were calculated as the number of deliveries per 1,000 total deliveries to determine if there were any identifiable changes in the rate of the outcomes over the 12-year study period. To assess the effect of maternal HIV infection on the probability of stillbirth and preterm birth, we conducted unadjusted and adjusted logistic regression models for each outcome at the 99% confidence level.

Variables selected for consideration in the logistic regression analysis were those that demonstrated strong associations (*p* < 0.001) with HIV infection and the birth outcome in question. We also tested several interactions with HIV status: maternal age, state (as a fixed effect), CCs, race, and SU. Because our analysis includes both HIV+ and HIV− women, ART use cannot be included as an analytic variable (since ART use is only present for one group).

Sensitivity analyses were performed excluding women over the age of 45 and resulted in no significant change. We also conducted a propensity score analysis (propensity to be HIV+); however, the results did not differ substantially. All statistical analyses were performed using STATA^®^ 17.0 distributed by StataCorp LLC. This study, and the waiver of informed consent, was reviewed and approved by the Brown University Institutional Review Board.

## Results

### Comparing HIV+ and HIV− pregnancies

We identified 7,696,836 deliveries born to 5,750,885 unique women. Of the deliveries, 33,078 were to HIV+ and 7,663,758 were to HIV− mothers. There were statistically significant differences (*p* < 0.001) between HIV+ and negative mothers for every characteristic examined (Table 1). The mean age for HIV+ women was 27 (standard deviation [SD]: 6.4) and 26 (SD: 6.2) for HIV−.

**Table 1.** Beneficiary Characteristics, by Human Immunodeficiency Virus Status

Total *n* = 7,696,836	HIV+ 33,078	HIV− 7,663,758	*p*
Outcomes			
Stillbirth	290 (0.9%)	51,323 (0.7%)	<0.001
Preterm birth	2,656 (8%)	507,990 (6.6%)	<0.001
Age groups			
Mean age (SD)	27 (6.4)	26 (6.2)	
18–24	13,249 (40.1%)	3,526,280 (46%)	<0.001
25–34	15,278 (46.2%)	3,287,461 (42.9%)	
35+	4,551 (13.8%)	850,017 (11.1%)	
Race/ethnicity			
White	10,013 (30.3%)	2,521,555 (32.9%)	<0.001
Black	10,968 (33.2%)	1,688,336 (22%)	
Hispanic	7,639 (23.1%)	2,584,221 (33.7%)	
Other	3,426 (10.4%)	616,188 (8%)	
Unknown	1,032 (3.1%)	253,458 (3.3%)	
Year			
2001	1,506 (4.6%)	471,870 (6.2%)	<0.001
2002	1,684 (5.1%)	518,650 (6.8%)	
2003	2,063 (6.2%)	556,325 (7.3%)	
2004	2,201 (6.7%)	600,657 (7.8%)	
2005	2,498 (7.6%)	638,927 (8.3%)	
2006	2,557 (7.7%)	666,828 (8.7%)	
2007	2,787 (8.4%)	675,841 (8.8%)	
2008	3,077 (9.3%)	688,729 (9.0%)	
2009	3,422 (10.3%)	700,828 (9.1%)	
2010	3,495 (10.6%)	679,641 (8.9%)	
2011	3,777 (11.4%)	718,874 (9.4%)	
2012	4,011 (12.1%)	746,588 (9.7%)	
State			
CA	2,205 (6.7%)	869,382 (11.3%)	<0.001
FL	4,078 (12.3%)	665,605 (8.7%)	
GA	2,471 (7.5%)	651,204 (8.5%)	
IL	2,914 (8.8%)	603,279 (7.9%)	
LA	1,584 (4.8%)	311,819 (4.1%)	
MA	515 (1.6%)	103,097 (1.3%)	
MD	817 (2.5%)	203,903 (2.7%)	
NC	2,205 (6.7%)	582,579 (7.6%)	
NJ	343 (1%)	124,247 (1.6%)	
NY	8,029 (24.3%)	1,146,419 (15.0%)	
OH	868 (2.6%)	244,936 (3.2%)	
PA	659 (2%)	165,179 (2.2%)	
TX	5,499 (16.6%)	1,742,350 (22.7%)	
VA	891 (2.7%)	249,759 (3.3%)	
Location			
Rural residence	2,313 (7%)	1,217,938 (15.9%)	<0.001
Basis of eligibility			
Adult	19,410 (58.7%)	4,491,408 (58.6%)	<0.001
Poverty	10,365 (31.3%)	2,758,318 (36%)	
Other	3,303 (10%)	414,032 (5.4%)	
CCs			
0 CC	2,527 (7.6%)	5,217,874 (68.1%)	<0.001
1 CC	7,215 (21.8%)	535,848 (7%)	
2 CCs	3,806 (11.5%)	569,632 (7.4%)	
3 CCs	4,092 (12.4%)	431,334 (5.6%)	
4 CCs	3,195 (9.7%)	293,998 (3.8%)	
5+ CCs	12,243 (37%)	615,072 (8%)	
Medicaid coverage			
Other	13,172 (39.8%)	2,441,874 (31.9%)	<0.001
FFS	19,906 (60.2%)	5,221,884 (68.1%)	
Prenatal visits			
No visits	13,911 (42.1%)	4,204,884 (54.9%)	<0.001
1–3 visits	7,681 (23.2%)	1,673,276 (21.8%)	
4–6 visits	4,415 (13.3%)	743,288 (9.7%)	
7+ visits	7,071 (21.4%)	1,042,310 (13.6%)	
SU			
Any SU	8,465 (25.6%)	585,654 (7.6%)	<0.001
Tobacco	5,232 (15.8%)	577,942 (7.5%)	<0.001

CCs, comorbid conditions; FFS, fee for service; HIV, human immunodeficiency virus; ; SD, standard deviation; SU, substance use; HIV+, HIV-positive; HIV–, HIV-negative.

HIV+ women were older (40.1% vs. 46%, respectively, in the 18- to 25-year age range), more often black (33.2% vs. 22%), less often Hispanic (23.1% vs. 33.7%), less likely to live in characteristically rural areas (7% vs. 15.9%), more likely to have one or more CCs (92.4% vs. 31.9%), less likely to have no prenatal visits (42.1% vs. 54.9%), and more likely to have an SU disorder (25.6% vs. 7.6%). HIV+ mothers were more likely to have stillbirth (0.9% vs. 0.7%) and preterm birth (8% vs. 6.6%). There were differences by state, for example 24.3% of the HIV+, but only 15% of the HIV−, pregnancies were from NY.

### Unadjusted and adjusted analyses of stillbirth

The unadjusted odds ratio (uOR) of stillbirth for HIV+ compared with HIV− mothers was 1.31 (Table 2, 99% CI: 1.12–1.53, *p* < 0.01). After adjusting for covariates, the adjusted odds ratio (aOR) was no longer statistically significant (aOR: 1.05). In the unadjusted analysis, older age, black race, rural residence, more CCs, an increased number of prenatal visits, SU disorder, and smoking were all associated with increased odds of stillbirth. In the adjusted analysis, most remained statistically significant, although generally with lower ORs.

**Table 2.** Unadjusted and Adjusted Analyses of Stillbirth

	Stillbirth			
	uOR	99% CI	aOR	99% CI
HIV+	1.31^**^	1.12–1.53)	1.05	0.90–1.23)
Age groups (ref: 18–24)				
25–34	1.08^**^	1.06–1.11)	1.18^**^	1.15–1.21)
35+	1.50^**^	1.45–1.55)	1.67^**^	1.61–1.73)
Race/ethnicity (ref: white)				
Black	1.90^**^	1.85–1.96)	1.99^**^	1.94–2.05)
Hispanic	0.88^**^	0.86–0.91)	1	0.97–1.04)
Other	0.93^**^	0.89–0.98)	1.01	0.96–1.06)
Unknown	1.18^**^	1.10–1.26)	1.21^**^	1.13–1.30)
Years (ref: 2001–2004)				
2005–2008	0.97^*^	0.94–1.00)	0.94^**^	0.91–0.96)
2009–2012	1.01	0.99–1.04)	0.92^**^	0.89–0.95)
Location				
Rural residence	1.04^*^	1.01–1.07)	1	0.97–1.03)
Basis of eligibility (ref: adult)				
Poverty	1.43^**^	1.40–1.47)	1.53^**^	1.49–1.57)
Other	1.48^**^	1.41–1.55)	1.43^**^	1.36–1.50)
CCs (ref: 0 CC)				
1 CC	1.19^**^	1.14–1.25)	1.11^**^	1.06–1.17)
2 CCs	1.16^**^	1.11–1.21)	1.08^**^	1.04–1.13)
3 CCs	1.20^**^	1.15–1.26)	1.12^**^	1.06–1.17)
4 CCs	1.23^**^	1.16–1.30)	1.14^**^	1.08–1.21)
5+ CCs	1.37^**^	1.32–1.42)	1.25^**^	1.20–1.31)
Medicaid coverage (ref: other)				
FFS	0.97^*^	0.95–1.00)	1.05^**^	1.02–1.08)
Prenatal visits (ref: 0 visits)				
1–3 visits	1.12^**^	1.09–1.15)	1.04^**^	1.01–1.07)
4–6 visits	1.16^**^	1.12–1.21)	1.05^**^	1.01–1.09)
7+ visits	1.15^**^	1.11–1.19)	1.01	0.98–1.05)
SU				
Any SU	1.24^**^	1.19–1.29)	1.04^*^	1.01–1.09)
Tobacco	1.16^**^	1.12–1.21)	0.99	0.95–1.04)

This analysis includes state fixed effects.

Robust CI in parentheses (^**^p < 0.01 and ^*^p < 0.05).

aOR, adjusted odds ratio; CI, confidence interval; uOR, unadjusted odds ratio.

Black race was strongly associated with stillbirth in the adjusted analysis (aOR: 1.99) when compared with white race. Hispanic race was not associated with stillbirth. Compared with adult, having a BOE for Medicaid of poverty (aOR: 1.53) or other (aOR: 1.43) was associated with stillbirth. A greater number of CCs were associated with stillbirth (*e.g.*, 5+ conditions compared with none, aOR: 1.25), as were any SU (aOR: 1.04). The only unexpected finding was that prenatal care was associated with increased risk of stillbirth (aOR 1.04). None of the interactions that we tested were statistically significant in this model.

### Unadjusted and adjusted analyses of preterm birth

The uOR of preterm birth for HIV+ compared with HIV− mothers was 1.23 (Table 3). After adjustment, the aOR was not statistically significant (aOR: 1.01). As with the stillbirth analysis, in the unadjusted analysis, older age, black race, rural residence, more CCs, more prenatal visits, an SU disorder, and smoking were all associated with increased odds of preterm birth. Similarly, in the adjusted analysis, most remained statistically significant, although generally with lower aORs.

**Table 3.** Unadjusted and Adjusted Analyses of Preterm Birth

	Preterm birth			
	uOR	99% CI	aOR	99% CI
HIV+	1.23^**^	1.17–1.30)	1.01	0.96–1.07)
Age groups (ref: 18–24)				
25–34	0.95^**^	0.94–0.95)	1.01^**^	1.00–1.02)
35+	1.06^**^	1.05–1.08)	1.16^**^	1.14–1.17)
Race/ethnicity (ref: white)				
Black	1.53^**^	1.51–1.54)	1.51^**^	1.49–1.52)
Hispanic	0.78^**^	0.78–0.79)	0.96^**^	0.95–0.97)
Other	0.97^**^	0.96–0.98)	1.04^**^	1.03–1.06)
Unknown	0.94^**^	0.91–0.96)	1.01	0.98–1.03)
Years (ref: 2001–2004)				
2005–2008	0.98^**^	0.97–0.99)	0.97^**^	0.96–0.98)
2009–2012	0.97^**^	0.96–0.98)	0.94^**^	0.93–0.95)
Location				
Rural residence	1.07	1.06–1.08)	1	0.99–1.01)
Basis of eligibility (ref: adult)				
Poverty	1.15^**^	1.14–1.15)	1.03^**^	1.02–1.04)
Other	1.33^**^	1.32–1.35)	1.20^**^	1.18–1.22)
CCs (ref: 0 CC)				
1 CC	1.24^**^	1.22–1.25)	1.12^**^	1.10–1.14)
2 CCs	1.19^**^	1.18–1.20)	1.09^**^	1.08–1.11)
3 CCs	1.25^**^	1.24–1.28)	1.12^**^	1.10–1.14)
4 CCs	1.30^**^	1.28–1.32)	1.16^**^	1.13–1.18)
5+ CCs	1.40^**^	1.39–1.42)	1.21^**^	1.19–1.23)
Medicaid coverage (ref: other)				
FFS	0.93^**^	0.92–0.94)	0.98^**^	0.97–0.99)
Prenatal visits (ref: 0 visits)				
1–3 visits	1.02^**^	1.01–1.03)	0.98^**^	0.97–0.99)
4–6 visits	1.06^**^	1.05–1.07)	0.98^**^	0.96–0.99)
7+ visits	1.07^**^	1.06–1.08)	0.97^**^	0.96–0.98)
SU				
Any SU	1.32^**^	1.31–1.34)	1.09^**^	1.08–1.11)
Tobacco	1.27^**^	1.25–1.29)	1.03^**^	1.02–1.05)

This analysis includes state fixed effects.

Robust CI in parentheses (^**^p < 0.01 and ^*^p < 0.05).

Compared with white race, black race was strongly associated with preterm birth (aOR: 1.51). Hispanic race was associated with a slightly lower odds of preterm birth (aOR: 0.96). In comparison with none, a greater number of CCs were associated with preterm birth (*e.g.*, 5+ conditions, aOR 1.21), as were any SU (aOR: 1.09). None of the interactions that we tested were statistically significant in this model.

## Discussion

In this 12-year nationally representative sample from the Medicaid program in the United States, we found no significant association between maternal HIV infection and either stillbirth or preterm birth. In both cases the aOR was very close to 1.0 (aORs of 1.05 and 1.01, respectively), and in both cases the 99% confidence intervals were relatively narrow, although they were tighter for preterm birth (aOR: 1.01, 99% CI: 0.96–1.07) than for stillbirth (aOR: 1.05, 99% CI: 0.90–1.23).

The literature on the association between HIV and stillbirth varies by time period covered, the type of data examined, the analytic methods used, and the potential confounders available. US studies^6,32^ and several studies from the developing world^14,15,20,33,34^ suggest a positive association between HIV and increased rates of stillbirth. A meta-analysis published in 1998 found no association with the adverse outcome in developed countries,^8^ and analyses that utilize data from the era of modern ART (the late 1990s and onward), tend to find no association.^11^

Our data are consistent with these more recent studies from developed countries. Studies that report a positive association tend to have limited power due to smaller sample sizes, vary in geographical and study location (*i.e.*, countries outside of the United States, single study site, and single US state), wide confidence intervals, and often do not have the ability to adjust for key potential confounders such as quality of prenatal care and maternal SU.

The prior literature assessing the relationship between HIV and preterm birth, although more abundant, is similarly diverse. Increased odds of preterm birth are reported among studies that compare HIV− and HIV+ women, before^7,19^ and after^6,21,27^ the modern ART era. Xiao et al., a meta-analysis that includes more recent studies, reports a pooled odds ratio of 1.56. One US article studied deliveries at two tertiary care centers between 2000 and 2007 found an adjusted OR of preterm birth for mothers with HIV of 2.27.^27^ Two studies that used the US National Inpatient Sample found adjusted ORs of 1.34^6^ (for 2003) and 1.57.^28^

HIV association with preterm birth is also observed in US studies with no HIV comparator^19,35^ including several studies adjusting for ART^6,21,36^ and those that did not.^20^ A few international studies did not find an association between HIV infection and preterm birth, and our results are consistent with these studies.^26,37,38^ Other studies from developing countries have reported positive associations.^14,15,22–24^ Similar to stillbirth literature, the positive studies tend to have limitations on power, study single sites or states, and are unable to control for key potential confounders.

Our analyses show how important adjustment for potential confounders is in understanding the relationship between maternal HIV infection and the two perinatal outcomes we studied. Multiple confounders were statistically significant in both models and tended to affect the aORs in similar ways. In both models older maternal age was associated with adverse outcomes, as has been shown previously.^29,30,39–41^ In both models, calendar year was associated with outcomes, with more recent years showing better outcomes. We are not aware of other analyses that show this finding. Explanations could include improving care for pregnant women broadly, increasing use of ART, or the selection of healthier women into the sample over time.

In both models, maternal chronic conditions were associated with worse outcomes, which is consistent with other literature.^42,43^ There was a weak but generally monotonic relationship between the number of chronic conditions and worse outcomes. Prenatal care was associated with slightly lower adjusted odds of preterm birth, but slightly higher odds of stillbirth.

The latter finding was unexpected, but could be a type of confounding by indication, that is, mothers with HIV could have health risks not captured by our covariates, causing them to seek out or receive more prenatal care. For example, an abnormal ultrasound might be associated with both more intensive prenatal care and a higher risk of an adverse outcome. We also found that substance abuse disorder was associated with a small increase in both stillbirth and preterm birth.

The strongest finding, which was consistent across the models, was the association between black race and poor birth outcomes, with aORs of 1.99 and 1.51 for black compared with white race in the stillbirth and preterm birth models, respectively. This relationship between black race and poor outcomes for pregnant women has been extensively documented previously,^40,41^ including in studies of the Medicaid program. This finding underlines the need to understand the social, economic, and medical inequities between black and white mothers and within Medicaid overall.

We view racism and differences in social conditions as being at root of these health inequities, and that, as such, racism and social conditions are fundamental causes of stillbirth and prematurity in the United States.^44,45^ This argument asserts that racism, interpersonal, institutional, or internalized, is a mechanism by which racial categorizations have clinical consequences.^46^ Evidence from our study supports this assertion—racial differences remain even after accounting for variables such as CCs, poverty, and SU. The causal factors driving these disparities are multifaceted and cannot be explained with the variables available in administrative claims data.

At least three considerations may help explain why HIV infection is not associated with stillbirth or preterm birth. The first consideration is the selection bias that may be present with our experimental design. This is an observational trial, and WLHIV who become pregnant are likely to be healthier than WLHIV who do not or cannot become pregnant. Should this selection bias exist, it would imply that our findings could be generalized to healthier WLHIV.

We cannot determine whether this selection bias is present in our data. Second, there are clearly biological factors that operate in the placenta—even in the absence of maternal ART use—to protect developing fetuses from the deleterious effects of maternal viremia.^8,47,48^ Finally, WLHIV have improved access to care, are living longer, and having healthier pregnancies. Current treatment for HIV and effective ART regimens reduces the risk for MTCT and adverse pregnancy complications.^8,49^ In a separate analysis we are examining the impact of ART on stillbirth and preterm birth in the women in our sample with HIV infection.

This study has several limitations. First, administrative claims databases have well-described limitations.^50–52^ They cannot capture important information such as clinical data (*e.g.*, ultrasound, amniocentesis results, viral load, or CD4 counts), individual risk behaviors, the stage of pregnancy at which outcomes occurred (*e.g.*, early vs. late), or the personal experiences of the women within our study sample.

Using inpatient and outpatient administrative claims data we are unable to assess the impact or quality of prenatal care, only present an estimate of the number of visits before delivery. Second, there may have been WLHIV that we could not identify using our HIV case finding algorithm; however, this misclassification bias would only cause a bias toward the null, which if corrected would only make our negative findings stronger.

Third, although our sampling strategy captured states that have ∼75% of the cases in the United States, our findings may not be generalizable to states that we did not study. Fourth, our findings may not be generalizable to women in the United States with commercial insurance. Fifth, our findings may not be generalizable to other developed countries, or to the developing world. Sixth, our analyses do not account for ART adherence or persistence and ART regimen in HIV+ mothers. Finally, we are unable to capture resources that Medicaid beneficiaries are receiving in addition to what is mentioned in the claims data (*e.g.*, participation in Ryan White Programs and access to free clinics).

Despite these limitations, our study has important strengths. To our knowledge, it is the first study to utilize a nationally representative sample to assess both stillbirth and preterm birth among HIV− and HIV+ mothers on Medicaid. The large sample size increases the power with which we could adequately detect any differences in outcomes according to HIV status. Notably, it is a strength that our study adjusts for state fixed effects. We assess multiple states in our analyses and the use of Medicaid administrative claims allowed us to adjust for a wide range of potential confounders, such as number of prenatal care visits; those of which are highly representative of the HIV+ population in the United States.

## Conclusions

In conclusion, this article uses a nationally representative Medicaid population from the era of modern ART to show that maternal HIV infection is not significantly associated with increased rates of stillbirth or preterm birth. For clinicians in the United States, an important implication of this research is that high-quality prenatal and perinatal care, including the use of ART during pregnancy, which has been a recommendation since 2008,^53^ should produce pregnancy outcomes similar to those of women who do not have HIV.

The role of ART use in the pregnancy outcomes of the WLHIV that we studied here is the topic of ongoing analysis. For medical researchers and social scientists, it is imperative that we understand and eliminate the clinical, social, and contextual factors that are responsible for the strong race findings that we observe. Additional policy strategies are needed within Medicaid to address and eliminate the disparities that disproportionately affect black women to improve their outcomes, such as expanding Medicaid coverage in the postpartum period and investing in social determinants of health (*i.e.*, transportation and housing).

## Abbreviations Used

Advance-CTR: advance clinical and translational research
aOR: adjusted odds ratio
ART: antiretroviral therapy
BOE: basis of eligibility
CCs: comorbid conditions
CHIP: Children's Health Insurance Program
CI: confidence interval
CPT: Current Procedural Terminology
FFS: fee for service
HIV: human immunodeficiency virus
HIV+: HIV-positive
HIV−: HIV-negative
ICD-9-CM: International Classification of Diseases, Ninth Revision, Clinical Modification
IP: Inpatient Hospital
MAX: Medicaid Analytic eXtract
MMIS: Medicaid Management Information System
MTCT: mother to child transmission
OT: other therapy
PS: personal summary
SD: standard deviation
SU: substance use
uOR: unadjusted odds ratio
WLHIV: women living with human immunodeficiency virus
